# Novel MUC1 Aptamer Selectively Delivers Cytotoxic Agent to Cancer Cells In Vitro

**DOI:** 10.1371/journal.pone.0031970

**Published:** 2012-02-22

**Authors:** Yan Hu, Jinhong Duan, Qimin Zhan, Fengdan Wang, Xin Lu, Xian-Da Yang

**Affiliations:** 1 Institute of Basic Medical Sciences, Chinese Academy of Medical Sciences and Peking Union Medical College, Beijing, China; 2 Cancer Institute and Hospital, Chinese Academy of Medical Sciences and Peking Union Medical College, Beijing, China; 3 Peking Union Medical College Hospital, Chinese Academy of Medical Sciences and Peking Union Medical College, Beijing, China; Wayne State University School of Medicine, United States of America

## Abstract

Chemotherapy is a primary treatment for cancer, but its efficacy is often limited by the adverse effects of cytotoxic agents. Targeted drug delivery may reduce the non-specific toxicity of chemotherapy by selectively directing anticancer drugs to tumor cells. MUC1 protein is an attractive target for tumor-specific drug delivery owning to its overexpression in most adenocarcinomas. In this study, a novel MUC1 aptamer is exploited as the targeting ligand for carrying doxorubicin (Dox) to cancer cells. We developed an 86-base DNA aptamer (MA3) that bound to a peptide epitope of MUC1 with a *K*_d_ of 38.3 nM and minimal cross reactivity to albumin. Using A549 lung cancer and MCF-7 breast cancer cells as MUC1-expressing models, MA3 was found to preferentially bind to MUC1-positive but not MUC1-negative cells. An aptamer-doxorubicin complex (Apt-Dox) was formulated by intercalating doxorubicin into the DNA structure of MA3. Apt-Dox was found capable of carrying doxorubicin into MUC1-positive tumor cells, while significantly reducing the drug intake by MUC1-negative cells. Moreover, Apt-Dox retained the efficacy of doxorubicin against MUC1-positive tumor cells, but lowered the toxicity to MUC1-negative cells (P<0.01). The results suggest that the MUC1 aptamer may have potential utility as a targeting ligand for selective delivery of cytotoxic agent to MUC1-expressing tumors.

## Introduction

Chemotherapy is an essential treatment for cancer, especially for late-stage metastatic disease. However, the efficacy of chemotherapy is often limited by the adverse effects of cytotoxic agents that are employed in most therapeutic regimens. A major problem associated with cytotoxic drugs is that they cause damages to both cancer cells and normal tissue, generating serious adverse effects that often limit the intensity and the duration of chemotherapy. Consequently, it is frequently difficult for cytotoxic drugs to eliminate all cancer cells within the body, resulting in treatment failure and poor prognosis. Such failure underscores the need to develop increasingly potent therapy with reduced toxicity. One approach to achieve the goal is targeted cancer therapy, in which anticancer drugs are selectively delivered to cancer cells, so that the cytotoxicity against tumor is enhanced while the adverse effects reduced. Targeted cancer therapy holds promise in improving anticancer efficacy. It has been reported that aptamer-guided carriers containing paclitaxel can significantly improve the efficacy against prostate cancer in animal models [1]. Monoclonal antibodies have also been covalently linked to drugs for targeted cancer therapy [2]. A study of trastuzumab emtansine (T-DM1), a conjugate of the humanized anti-HER2 antibody and a chemical drug, is currently in phase III clinical trial [3].

A typical targeted drug delivery system usually consists of an anticancer drug and a targeting ligand, which can specifically binds to tumor markers that abundantly express in cancer cells [4]. An ideal tumor marker for targeted therapy should be a membrane protein that is overexpressed on the surface of cancer cells, with relatively low expression in normal tissue. MUC1 is a well-characterized large transmembrane glycoprotein that may potentially serve as the target for anticancer therapy. Its expression is increased by at least 10-fold in most malignant adenocarcinomas, including breast cancer, lung cancer, and colon cancer, making it an attractive tumor marker for targeted therapy [5]. Besides the tumor marker, the tumor-targeting ligand is also an important element for targeted cancer therapy. An ideal targeting ligand should have high binding affinity for the tumor marker, with good specificity and low immunogenicity [6]. Lately, novel targeting agents, including aptamers [7], short peptides [8] and other small molecules [9], have become the new generation targeting molecules. Aptamers are single-strand oligonucleotides that can bind to target molecules with high affinity and specificity. Comparing to monoclonal antibodies, aptamers possess distinctive advantages as targeting ligand: high affinity for binding to most molecules, limited synthesis cost, low-immunogenicity, and small size that allows it to penetrate solid tumors [10]. Due to these advantages, aptamers have been employed as novel targeting ligands in drug delivery systems against prostate cancer [11], [12], [13] and leukemia [14]. For the tumor marker of MUC1, Ferreira et al have developed several aptamers that could bind to the MUC1-positive tumor cells [15]. It has also been shown that the MUC1 aptamers could be employed to selectively deliver phototherapy agent to cancer cells *in vitro* [16].

Cytotoxic drugs are the major components of most chemotherapy regimens for cancer treatment. It is therefore important to explore whether MUC1 aptamer can be used directly to selectively deliver cytotoxic agent to cancer cells. So far, however, no such study has been reported in literature. Here in this study, we developed a novel MUC1 aptamer, and evaluated its capacity for delivering doxorubicin to cancer cells *in vitro*. Specifically, we selected an 86-base DNA aptamer (termed MA3) against a peptide epitope of MUC1, and evaluated its binding affinity to MUC1-positive and -negative cells. To explore the binding specificity of the aptamer, we also evaluated its binding to albumin, which is the most abundant protein in plasma, and may non-specifically bind to aptamers and interfere with their targeting function. Doxorubicin is one of the most widely used anticancer drugs, and can inhibit the proliferation of cancer cells through intercalating into the DNA structure in cell nuclei. An aptamer-doxorubicin complex (Apt-Dox) was formulated by incorporating doxorubicin into the aptamer structure of MA3. We now report that Apt-Dox can selectively deliver doxorubicin to MUC1-positive cells *in vitro*.

## Materials and Methods

### Reagents

Oligonucleotide primers were synthesized by Invitrogen (Shanghai China). Peptides of at least 95% purity were synthesized by SBS Genetech (Beijing China). Bovine serum albumin (BSA) was purchased from Tbdscience (Tianjin China). Monodispersed magnetic urea-formaldehyde microspheres were purchased from Baseline Chromtech (Tianjin China). Trypsin was purchased from Amresco (US). Streptavidin-coated magnetic beads were purchased from Promega (US). 1-Ethyl-3-(3-dimethyllaminopropyl)carbodiimide hydrochloride (EDC) was purchased from Sigma (US).

### Cell lines

Human breast cancer (MCF-7), human liver cancer (HepG2), human lung cancer (A549), and human normal liver cells (L02) were obtained from the Cell Center of Chinese Academy of Medical Sciences (Beijing, China). Cells were cultured in DMEM medium supplemented with 10% fetal bovine serum (FBS) and a mixture of penicillin/streptomycin. Cells were grown at 37°C in a humidified atmosphere with 5% of CO_2_. All experiments were performed on cells in the exponential growth phase.

### Immobilization of target on magnetic beads

The target for aptamer selection is a 9-AA peptides (peptide1) with the sequence of APDTRPAPG. The conjugation of the target to magnetic beads was accomplished via cross-linking of −COOH and −NH2. Two µg of peptide1 was mixed with 5×10∧5 carboxylated magnetic beads, and incubated with 200 µl EDC (40 mM) at room temperature with gentle stirring for 2 h. The magnetic beads were then washed for three times with Hanks buffer, and stored at 4°C. Similar method was employed to conjugate the beads with other substances, including BSA and a 29-AA long peptides with the sequence of GSTAPPAGHGVTSAPDTRPAPGPGSTAPP (peptide2).

### SELEX library and primers

A starting DNA library consisting of 86-mer oligonucleotides with central 40-base long randomized sequences was synthesized. The sequence of library is 5′AACCGCCCAAATCCCTAAGAGTC-N40-CACAGACACACTACACACGCACA3′, where N represents a randomized nucleotide of either A, G, C or T. An FITC-labeled 5′ primer (5′-FITC-AACCGCCCAAATCCCTAAGAGTC-3′) and a biotin-labeled 3′ primer (5′-B-TGTGCGTGTGTAGTGTGTCTGTG-3′) were used in the PCR for the synthesis of double-labeled, double-stranded DNA molecules, which was then mixed with streptavidin-coated magnetic beads for 10 min at room temperature. After denaturing in alkaline condition (0.1 M NaOH), the FITC-conjugated sense single-strand DNA (ssDNA) was separated from the biotin-labeled antisense ssDNA strand with streptavidin-coated magnetic beads and used for aptamer selection.

### In vitro selection of target-binding aptamers

The procedures of selection were as follows. The ssDNA pool (200 pmol) was first heated at 95°C for 5 min and then cooled immediately to 0°C in binding buffer (Hanks buffer) for 15 min before binding to target. To reduce background interference, 0.1 mg/ml salmon sperm DNA and 1 mg/ml of BSA were added to the binding buffer. In the initial selection round, the peptide1-coated beads were suspended in 200 µl of binding buffer containing 200 pmol of random ssDNA. After incubating the mixture at 37°C for 30 min with gentle shaking, the unbound oligonucleotides were removed by washing four times with 500 µl of binding buffer. Subsequently, bead-bound oligonucleotides mixtures were amplified by PCR with FITC- or biotin-labeled primers (25 cycles of 40 sec at 94°C, 30 sec at 65°C, 40 sec at 72°C, followed by 10 min at 72°C, the Taq polymerase and dNTPs were obtained from Takara). The ensuing dsDNA from PCR was separated into ssDNA via procedure described above. The selected sense ssDNA separated from the biotinylated antisense DNA strand by streptavidin-coated magnetic beads was used for the next round of SELEX. After several rounds of selection, the selected ssDNA pool was PCR-amplified using unmodified primers and cloned into Escherichia Coli with the TA cloning kit for DNA sequencing.

### Flow cytometry analysis

To monitor the enrichment of aptamer candidates after selection, the FITC-labeled ssDNA pool was incubated with peptide1-coated magnetic beads in 200 µl of selection buffer containing 10% FBS at 37°C for 30 min. The beads were washed twice with 0.5 ml of binding buffer and then suspended in 0.2 ml of binding buffer. The FITC fluorescence was determined with a FACScalibur cytometer (Accuri C6, US). The FITC-labeled randomized ssDNA library was used to generate the control signal.

The binding affinity of aptamers was determined by incubating peptide1-coated magnetic beads with varying concentrations of FITC-labeled aptamer in 200 µl of binding buffer at 37°C for 30 min. The beads were washed twice with 0.5 ml of binding buffer, suspended in 0.2 ml of binding buffer, and subjected to flow-cytometric analysis. The FITC-labeled unselected ssDNA library was used as a negative control to determine nonspecific binding. All of the experiments for binding assay were repeated three times. The mean fluorescence intensity of target labeled by aptamers was used to calculate for specific binding by subtracting the mean fluorescence intensity of nonspecific binding from unselected library DNA [17]. The equilibrium dissociation constants (*K*_d_) of the aptamer–peptide1 interaction were obtained by fitting the dependence of fluorescence intensity of specific binding on the concentration of the aptamers to the equation Y = B max X/(Kd+X).

To evaluate the specific binding of aptamers to target, the FITC-labeled aptamer was separately incubated with peptide1-, peptied2-, or BSA-coated magnetic beads. The beads were washed twice with 0.5 ml of binding buffer, suspended in 0.2 ml of binding buffer, and analyzed by flow cytometry. For assessing aptamer binding to cells, cells were scraped off the culture bottle and washed with Hanks buffer. The FITC-labeled aptamer was incubated with 10∧5 of either MCF-7, A549, HepG2 or L02 cells in binding buffer at 37°C for 30 min. Cells were then washed thrice with Hanks buffer and analyzed with flow cytometrey.

### Cells and siRNA transfection

A549 cells were plated in 6-well plates at 1–3×10^5^cells/well, grown in antibiotic-free media overnight, and transfected with MUC1 siRNA or control siRNA [18] using Lipofectamine 2000 in Opti-MEM I reduced serum medium according to the manufacturer's instructions (Invitrogen Life Technology, Inc.). After 72 hours, cells were harvested and stained with FITC labeled aptamer for flow cytometry assay, and cell lysates were prepared for immunoblot analysis.

### Western blot analysis

Lysates were prepared from subconfluent cells. Equal amounts of protein were separated by SDS-PAGE and transferred to nitrocellulose membranes. The immunoblots were probed with anti-MUC1 antibody. The immunocomplexes were detected with horseradish peroxidase-conjugated secondary antibodies and enhanced chemiluminescence (ECL).

### Loading of aptamer with doxorubicin

Aptamers were first heated at 95°C for 5 min and then cooled immediately to 0°C in water for 15 min. The aptamers were incubated in an aqueous solution of doxorubicin (3 nM) for 1 h in a black 96-well plate at various aptamer/dox molar ratios. The fluorescence spectrum of doxorubicin was then examined by a Synergy4 analyzer (λEx = 488 nm, λEm = 500–700 nm). FITC-labeled Apt-Dox or Apt-Dox were incubated with A549 cells in binding buffer at 37°C for 30 min. Cells were then washed thrice with Hanks buffer and analyzed with flow cytometrey.

### Cellular uptake of doxorubicin

The specific cellular uptake of Apt-Dox by MUC1-positive A549 cell was studied by confocal fluorescence scanning microscopy (Perkin Elmer Ultraview, US) and flow cytometry. HepG2 cell was used as a MUC1-negative control. Cells were allowed to adhere to a glass cover slip for 24 h. The cells were then incubated with 1.5 µM of Dox or Aptamer-Dox for 2 h at 37°C. After being washed twice with Hanks buffer, the cells were fixed with 4% formaldehyde for 10 min and analyzed by confocal fluorescence scanning microscopy.

For flow cytometry analysis, cells were scraped off from the culture bottle and washed twice with Hanks buffer. The cells were incubated with 1.5 µM Dox or Aptamer-Dox for 4 h at 37°C, and washed twice with Hanks buffer. The cells were then fixed with 4% formaldehyde for 10 min and analyzed with flow cytometriy.

### MTS cell viability assay

To evaluate the cytotoxicity of Apt-Dox or Dox against A549 and HepG2 cells, both cell lines were first grown in 96-well plates, and then co-incubated at 37°C with Apt-Dox, Dox, or aptamer at the concentration of 3 µM for 4 h. The cells were washed with Hanks buffer for two times, and cultured for a further 48 h. Afterwards, MTS assay (Promega, US) was used to determine the cell viability per standard protocol outlined by the manufacture.

### Statistics

Statistical analysis was performed using Statistical Analysis System (SAS, Version 9.2). One-way ANOVA with Fisher's least significant difference (LSD) post hoc comparisons at 99% confidence interval was used for statistical comparisons. All data are presented as a mean value with its standard deviation indicated (mean ± SD).

## Results

### Aptamer selection and characterization

**Figure 1:**
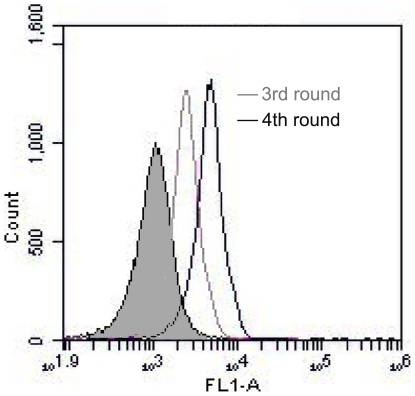
Flow cytometry monitoring of the enrichment of aptamers. Compared with the starting random DNA pool (shaded histogram), flow cytometry revealed an increase in fluorescence intensity of aptamers bound to the MUC1 peptide (APDTRPAPG) after the third (the gray curve) and forth (the black curve) rounds of selection.

The extracellular protein core of MUC1 is made up of variable number of a highly conserved tandem repeat sequence composed of 20 amino acids (TAPPAGHGVTSAPDTRPAPGPGS) [19]. Among the tandem repeat, the sequence APDTRPAPG had been identified as the most immunodominant peptide epitope [20], and was used as the target for MUC1 aptamer selection in prior research [15]. Here in this study, we also used this peptides as the target in our SELEX process. The target peptides were covalently conjugated to magnetic beads using EDC as the catalyst. During each round of selection, the beads were incubated with FITC-labeled ssDNA pool, and the enrichment of aptamers was monitored by flow cytometry. Compared with the random DNA from the library pool, an increasing amount of ssDNA bound to target-coated magnetic beads after each round of selection (Fig. 1). The aptamers were subsequently cloned, and 50 clones were analyzed for further characterization. Among these clones, one apatmer termed MA3 showed relatively high binding to the target MUC1 peptides. The DNA sequence of the aptamer MA3 is 5′AACCGCCCAAATCCCTAAGAGTCGGACTGCAACCTATGCTATCGTTGATGTCTGTCCAAGCAACACAGACACACTACACACGCACA3′.

**Figure 2:**
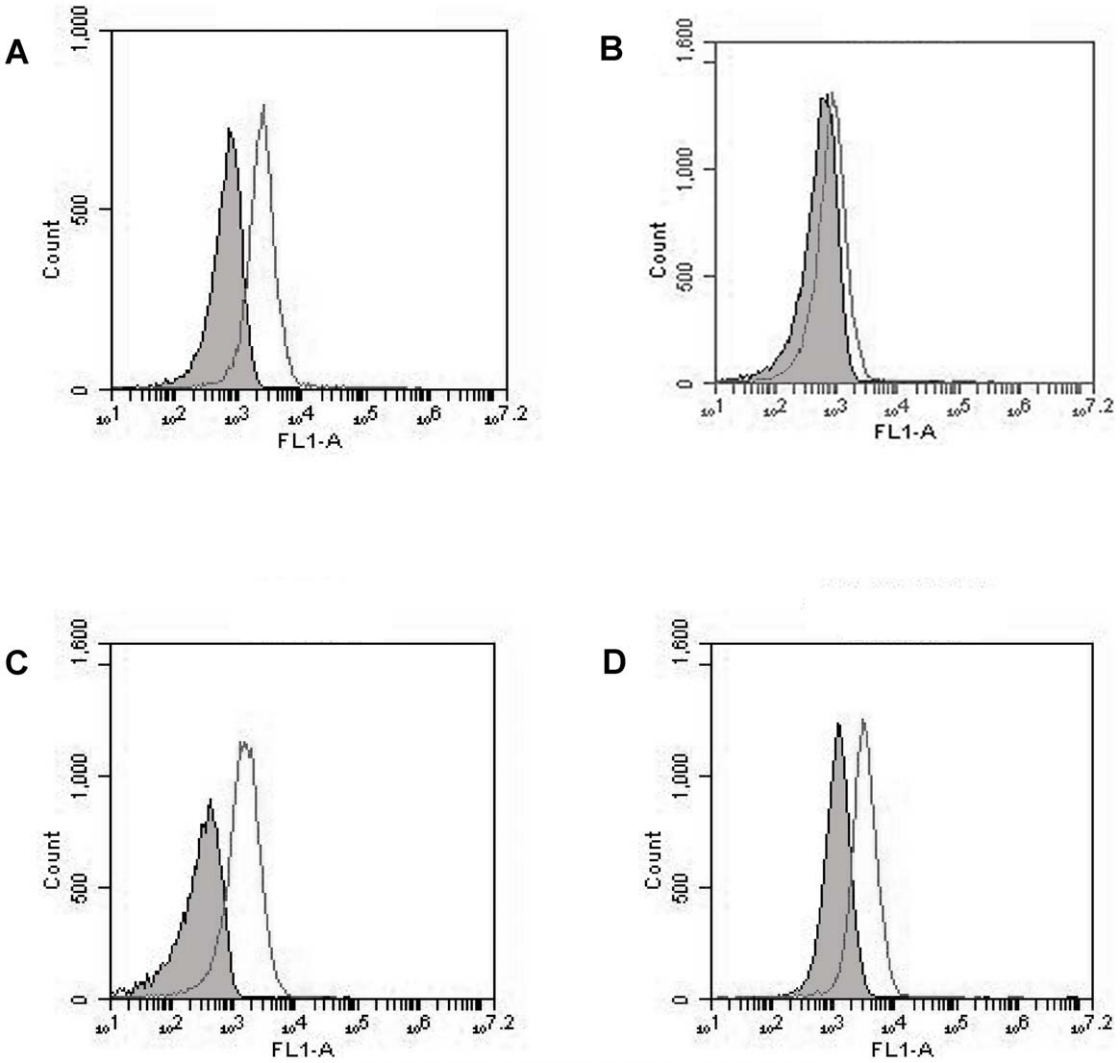
Flow cytometry assessment of the bindings to MUC1-peptide or BSA by FITC-labeled aptamers MA3 and S2.2. (A) MUC1-peptide beads treated with MA3. (B) BSA beads treated with MA3. (C) MUC1-peptide beads treated with S2.2. (D) BSA beads treated with S2.2. The filled histograms represent the control fluorescent signals generated by FITC-labeled random DNA from the library pool.

Binding specificity is important for evaluation of aptamer performance. Since albumin is the most abundant protein in blood, we examined the binding of the aptamer MA3 to albumin. Albumin or MUC1 peptides coated beads were incubated with FITC-labeled MA3, and analyzed by flow cytometry. Unselected random DNA from the library pool was used as control. As presented in Figure 2, the MA3 aptamer generated a significant binding to MUC1 peptides (Fig. 2A), but a relatively weak binding to BSA similar to that generated by random DNA (Fig. 2B). The results suggested that, between the MUC1 peptides and albumin, the aptamer MA3 exhibited a targeting specificity towards the former. As a comparison, similar experiments were conducted for another MUC1 aptamer, S2.2, which had the lowest *K*_d_ among all the published MUC1 aptamers [15], [16]. The results showed that while S2.2 could bind to MUC1 peptides (Fig. 2C), it also bound to albumin to a certain degree (Fig. 2D). The data indicated that, compared to S2.2, MA3 had a lower cross reactivity to albumin.

**Figure 3:**
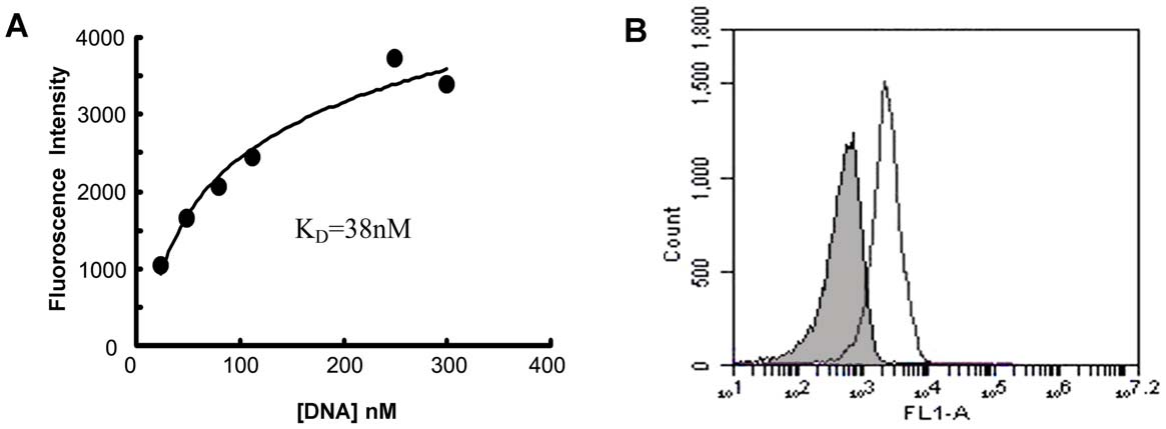
Evaluation of MA3's affinity to MUC1 structures. (A) Quantitative assay of the affinity between FITC-labeled MA3 and the MUC1 epitope (APDTRPAPG). (B) Flow cytometry analysis of binding between FITC-labeled MA3 and the 26-AA MUC1 peptide (GSTAPPAGHGVTSAPDTRPAPGPGSTAPP). The filled histogram is the control fluorescence background generated by FITC-labeled random DNA from the library pool.

To quantitatively evaluate the binding affinity of the aptamer to MUC1, beads coated with MUC1 peptides were incubated with FITC-labeled MA3 of various concentrations (Fig. 3A). Using non-linear regression analysis, the aptamer was found to have a *K*_d_ of 38.3 nM. To further evaluate whether the aptamer MA3 would bind to MUC1 structure, we also examined its binding to a 29-AA peptides (GSTAPPAGHGVTSAPDTRPAPGPGSTAPP) containing the entire tandem repeat sequence (TAPPAGHGVTSAPDTRPAPGPGS) that made up the extracellular MUC1 protein core. The 29-AA peptides were covalently conjugated to magnetic beads, which were incubated with FITC-labeled MA3 and analyzed by flow cytometry. FITC-labeled random DNA from the library pool was used as control. As shown in Fig. 3B, MA3 generated a fluorescence signal that was significantly more potent than random DNA, indicating that the aptamer could also bind to a structure containing the entire MUC1 tandem repeat sequence. Since the extracellular domain of MUC1 protein core is primarily made of the tandem repeats, it is possible that the aptamer MA3 may recognize the MUC1 structure exposed on the surface of MUC1-expressing tumor cells.

### MA3 aptamer selectively binds to MUC1-expressing tumor cells

**Figure 4:**
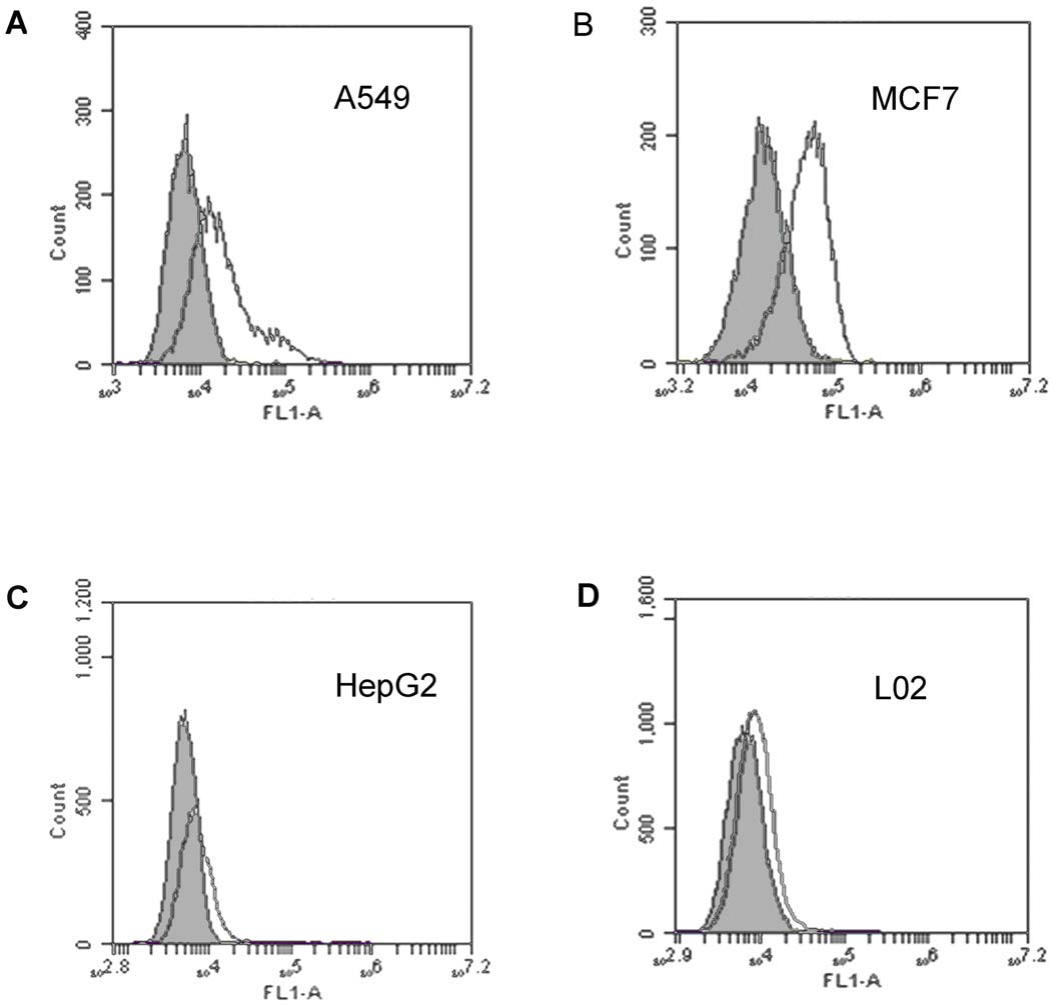
Flow cytometry evaluation of MA3's binding to MUC1-positive and -negative cells. The histograms were generated after incubating FITC-labeled MA3 with A549 (A), MCF7 (B), HepG2 (C), and L02 (D) cells, respectively. The filled histograms represent the control fluorescence signals generated by FITC-labeled random DNA from the library pool.

To evaluate whether the aptamer MA3 would bind to MUC1-expressing cancer cells, FITC-labeled MA3 was incubated with either the MUC1-positive (A549 or MCF7) [21], [22] or the MUC1-negative (HepG2 or L02) cell lines [21], [23]. The cells were later analyzed by flow cytometry, using the cells incubated with FITC-labeled random DNA as control. The flow cytometric profiles are presented in Figure 4. For MUC1-positive cell lines A549 and MCF7, MA3 treatment generated fluorescence signals that were significantly more potent than that of random DNA treatment (Fig. 4A and B). However, for MUC1-negative cell lines HepG2 and L02, MA3 treatment resulted in fluorescence signals that were similar to that of random DNA treatment (Fig. 4C and D). The results indicated that the aptamer MA3 could preferentially bind to MUC1-positive cancer cells, and that the aptamer might recognize the MUC1 structure on these cells.

### MUC1 expression influences the binding of the aptamer to tumor cells

**Figure 5:**
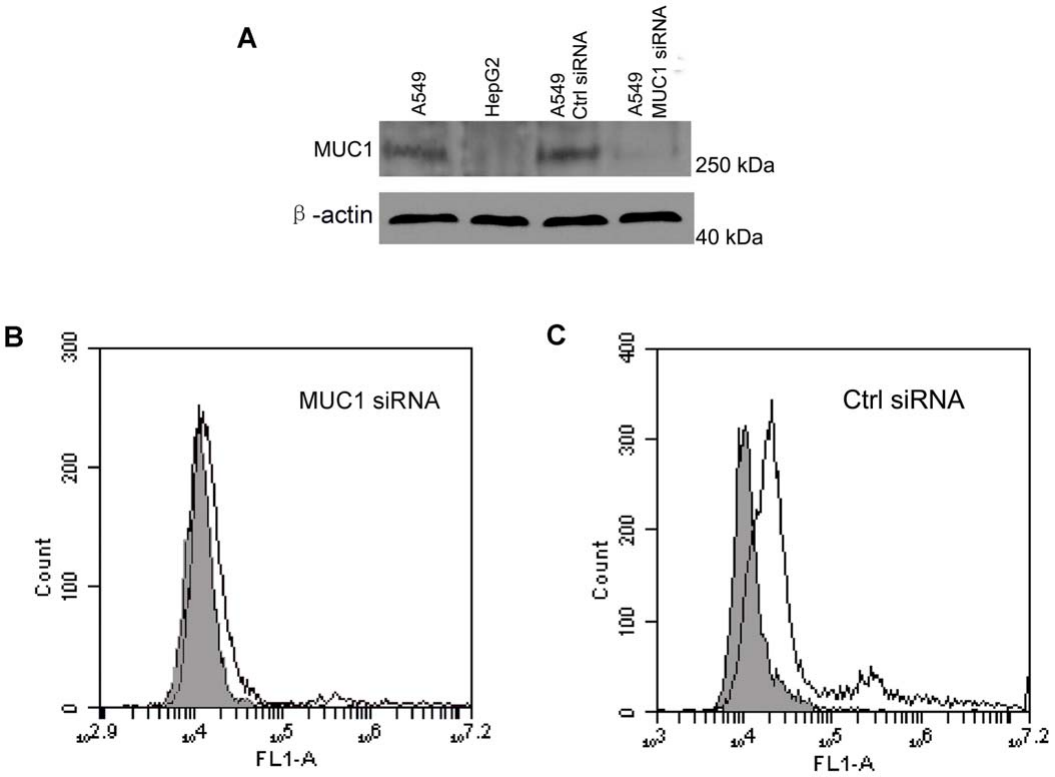
Western blot of MUC1 protein and siRNA interference experiments. (A) Western blots of protein extracts from A549 cells, HepG2 cells, A549 cells treated with control siRNA, and A549 cells treated with MUC1 siRNA, respectively. Actin was also blotted to serve as the control. (B and C) Flow cytometry evaluation of the apatamer's binding to A549 cells treated with MUC1 siRNA (B) or control siRNA (C). The filled histograms represent the control fluorescence signals generated by FITC-labeled random DNA from the library pool.

To further investigate the binding of the aptamer to MUC1-positive tumor cells, the expression of MUC1 protein in A549 and HepG2 cells were evaluated by western blot with anti-MUC1 antibody. As presented in Figure 5A, while A549 cells expressed MUC1 protein in ample amount, HepG2 cells did not, suggesting that A549 was indeed a MUC1-positive cell line and that HepG2 was a MUC1-negative cell line. The results are in agreement with multiple published studies, which extensively analyzed the expression of the MUC1 in various cell lines and clearly identified A549 as MUC1-positive and HepG2 as MUC1-negative cell line [18], [24].

To evaluate the influence of MUC1 expression on the aptamer's binding to MUC1-positive cells, we utilized MUC1 siRNA that had been previously shown capable of knocking down the MUC1 expression [18]. The siRNA was transfected into the MUC1-positive A549 cells, and was confirmed with western blot that it could down regulate the MUC1 expression (Fig. 5A). A549 cells were treated with either MUC1 siRNA or a control siRNA, incubated with FITC-labeled aptamer, and evaluated by flow cytometry. As presented in Figure 5B and C, the aptamer binding to the cells was significantly reduced after down-regulation of MUC1 expression (Fig. 5B), whereas the binding to cells treated with control siRNA were not affected (Fig. 5C). The results suggested that MUC1 expression on target cells influenced the aptamer's affinity to these cells, and that the loss of MUC1 protein could reduce the aptamer binding significantl*y*.

### Loading the aptamer with Dox

**Figure 6:**
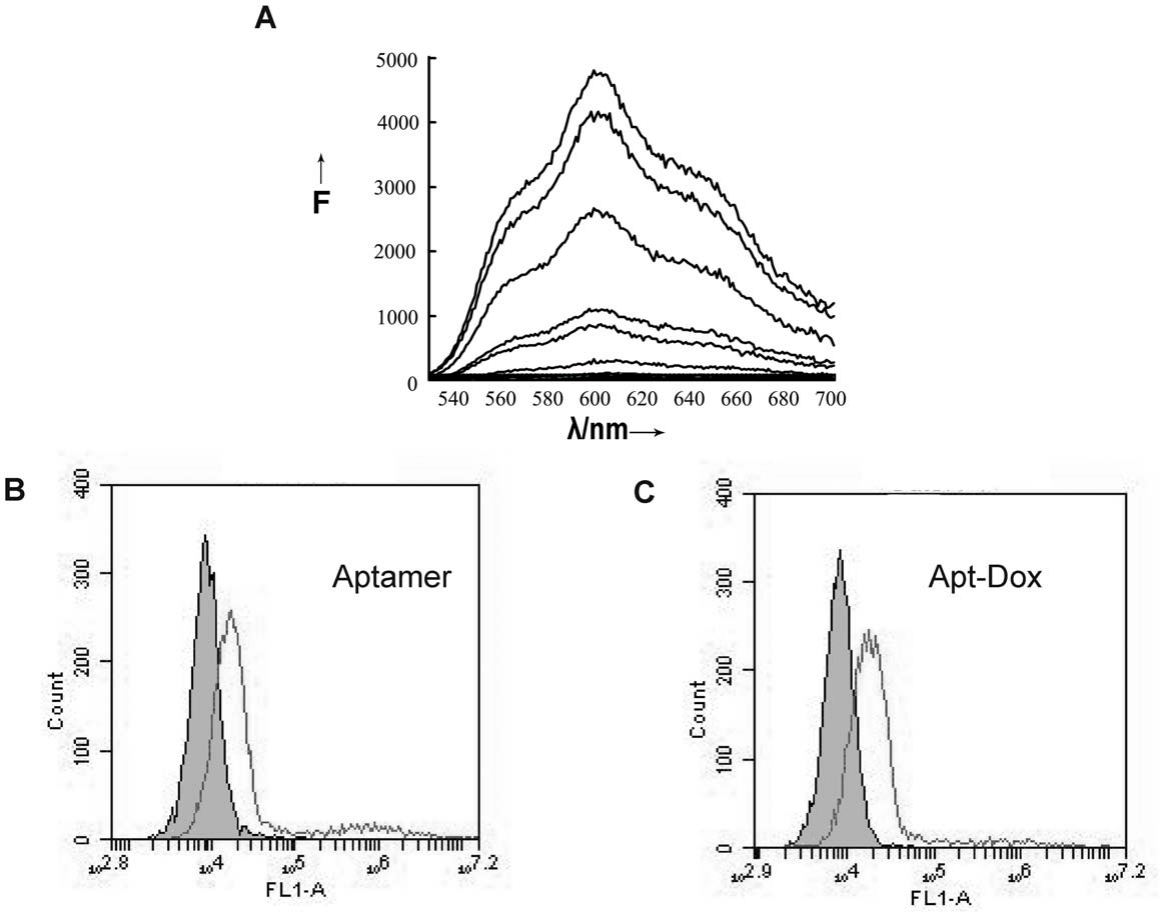
Fluorescence spectra of Apt-Dox and flow cytometry evaluation of Apt-Dox's binding to A549 cells. (A) Fluorescence spectra of doxorubicin solution mixed with increasing molar ratios of the MA3 aptamer (from top to bottom: 0, 0.001, 0.003, 0.005, 0.01, 0.03, 0.1, and 1). (B) A549 cells treated with FITC-labeled MA3 aptamer. (C) A549 cells treated with FITC-labeled Apt-Dox. The filled histograms represent the control fluorescence signals generated by FITC-labeled random DNA from the library pool.

In order to deliver Dox to MUC1-positive tumor cells, an aptamer-doxorubicin complex (Apt-Dox) was formed by intercalating doxorubicin into the DNA structure of the MA3 aptamer. To evaluate whether Dox was indeed incorporated into MA3, we made use of the phenomenon that the fluorescence from Dox would be quenched after intercalating into DNA [25]. Specifically, we carried out binding studies between MA3 and Dox, and employed fluorescence spectroscopy to assess the fluorescent signal generated by doxorubicin. As shown in Figure 6A, sequential decreases in the native fluorescence spectrum of Dox were observed when a fixed concentration of Dox was incubated with an increasing molar ratio of the MA3 aptamer. When the aptamer/dox molar ratio reached 0.1, the fluorescence spectrum of Dox was at the lowest level and did not change further, indicating that most dox had incorporated into the DNA structure of MA3 at this aptamer/dox ratio.

To investigate whether the intercalation of doxorubicin into aptamer would interfere with the aptamer's affinity to MUC1-positive tumor cells, the binding of Apt-Dox complex to A549 cells was evaluated with flow cytometry, and compared with that of MUC1 aptamer alone. As shown in Figure 6B and C, Apt-Dox complex (Fig. 6C) had an affinity to A549 cells that was similar to MUC1 aptamer alone (Fig. 6B). The results suggested that intercalation of doxorubicin into MUC1 aptamer did not significantly interfere with the binding of the aptamer to MUC1-positive tumor cells.

### Aptamers as vehicles for selective delivery of doxorubicin to MUC1 positive cancer cells

**Figure 7:**
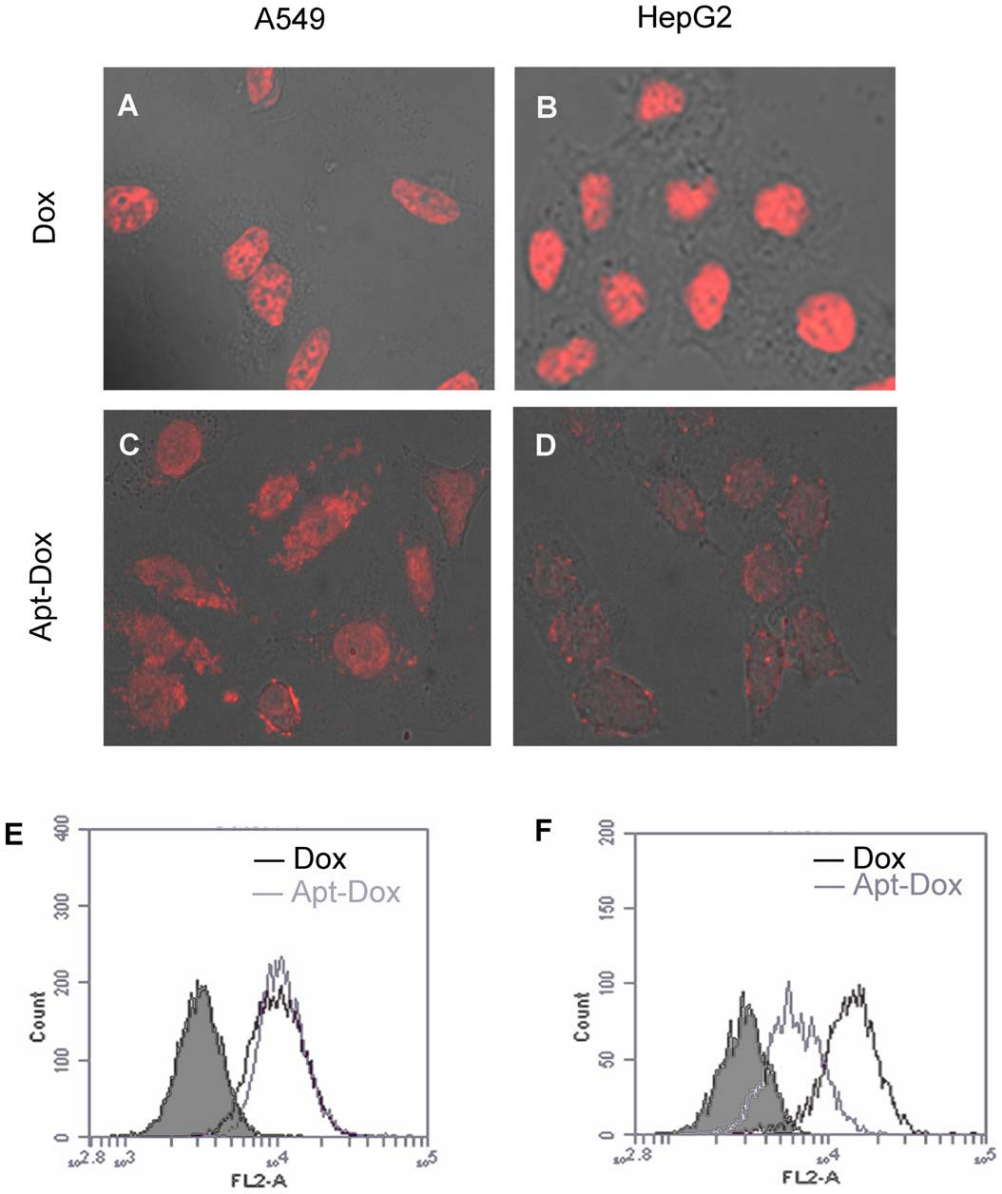
Uptake of doxorubicin by MUC1-positive (A549) and -negative (HepG2) cells. (A–D) Confocal laser scanning microscopy images of A549 and HepG2 cells after incubation with free doxorubicin (A and B) or Apt-Dox (C and D) for 2 hours. (E and F) Flow cytometry histogram profiles of A549 (E) and HepG2 cells (F) after incubation with either free doxorubicin (black curves) or Apt-Dox (gray curves). The filled histograms are the control signals generated by untreated cells.

The nonselective uptake of free Dox by both cancer and normal cells is a primary cause for its adverse effects against normal tissue. When Dox is intercalated into the DNA structure of the MUC1 aptamer (Apt-Dox), the complex may preferentially bind to MUC1-positive cancer cells. To test this postulate, the fluorescence properties of doxorubicin were utilized to evaluate the drug uptake by either MUC1-positive (A549) or MUC1-negative (HepG2) cells. The cells were incubated separately with free Dox or Apt-Dox and analyzed by confocal microscopy. The results showed that the red fluorescence from the drug was equally strong in the two cell lines treated with free Dox (Fig. 7A and B), indicating that the uptake of free Dox by these cells was non-selective. In contrast, when the cells were treated with Apt-Dox, the fluorescence in MUC1-positive A549 cells was significantly higher than that in MUC1-negative HepG2 cells (Fig. 7C and D), and that the uptake of the drug by HepG2 cells was markedly decreased. The results suggested that Apt-Dox exhibited cell-specificity and could be selectively taken up by MUC1-positive cells. Interestingly, the intracellular distribution of the drug in A549 cells was mainly limited to the nuclei with free Dox, but extended to cytoplasm in the setting of Apt-Dox, suggesting that the mechanisms for uptake of free Dox and Apt-Dox might be different.

To further study whether Apt-Dox could be selectively taken up by MUC1 positive cells, flow cytometry was also employed to monitor the fluorescence generated by doxorubicin after incubating the two cell lines with free Dox or Apt-Dox. For MUC1-positive A549 cells, the fluorescent signals generated by free Dox or Apt-Dox were similar (Fig. 7E); whereas for MUC1-negative HepG2 cells, the fluorescent signal generated by Apt-Dox was remarkably lower than that generated by free Dox (Fig. 7F). The results again suggested that Apt-Dox could be selectively taken up by MUC1 positive cancer cells. Taken together, our microscopy and flow cytometry data indicate that the aptamer MA3 may serve as a vehicle for targeted delivery of Dox to MUC1-positive cancer cells.

### Apt-Dox selectively reduced cytotoxicity to MUC1-negative cancer cells

**Figure 8:**
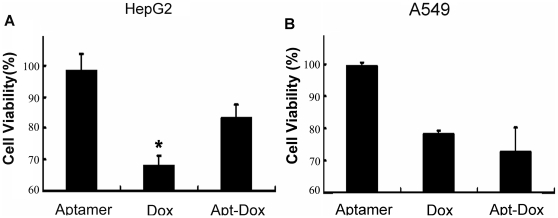
Cell viability assays after the cells were treated with aptamer, free Dox, or Apt-Dox for 4 hours. The HepG2 (A) and A549 (B) cells were evaluated with a standard MTS assay after 48 h of further incubation (mean±SD, n = 6).

Since the uptake of doxorubicin was reduced in MUC1-negative cells treated with Apt-Dox, it is possible that the cytotoxicity to these cells would also be decreased. To test the postulate, *in vitro* cytotoxicity caused by Apt-Dox or free Dox was compared in HepG2 and A549 cell lines. As presented in Figure 8A, for MUC1-negative HepG2 cells, the cytotoxicity generated by Apt-Dox was indeed decreased compared to that generated by free Dox (P<0.01). However, for MUC1-positive A549 cells, no difference was detected between the cytotoxicity generated by Apt-Dox or free Dox (Fig. 8B). The results suggest that Apt-Dox tends to reduce the damage to MUC1-negative cells while retaining the efficacy of the doxorubicin against MUC1-positive cells. It should be noted that aptamer alone was nontoxic towards both cell lines, indicating that the aptamer itself was relatively safe to the cells and that the cytotoxicity was mainly caused by doxorubicin.

## Discussion

The efficacy of chemotherapy is often limited by the adverse effects of cytotoxic agents that damage both cancer and normal cells. One strategy for reducing the adverse effects of chemotherapy is targeted drug delivery to cancer cells. MUC1 is considered a valuable target for ligand-guided anticancer chemotherapy due to its over-expression in most adenocarcinomas. Here in this study, using the SELEX technique and a peptide epitope of MUC1 as target, we developed a novel MUC1 aptamer (MA3) with a *K*_d_ of 38.3 nM. We found that MA3 could specifically bind to MUC1-positive cancer cells, with minimal cross reactivity to albumin (Fig. 1,2,3,4). Moreover, an aptamer-drug complex (Apt-Dox) was formed by intercalating doxorubicin into the DNA structure of the MA3 aptamer (Fig. 6). Importantly, Apt-Dox resulted in a selective uptake of doxorubicin into MUC1-positive cells while significantly reduced the drug intake by MUC1-negative cells (Fig. 7). In addition, Apt-Dox retained the efficacy of doxorubicin against MUC1-positive cells, while notably lowered the toxicity to MUC1-negative cells (Fig. 8). The results suggested that Apt-Dox could discriminate between target and non-target cells.

The extracellular domain of MUC1 is a glycoprotein consisting of a protein core and O-linked oligosaccharides [19]. The protein core contains variable number of a tandem repeat sequence composed of 20 amino acids (TAPPAGHGVTSAPDTRPAPGPGS). In normal tissue, the protein core of MUC1 is usually shielded by the saccharide chains; whereas in tumor cells, the protein core is exposed due to deficient glycosylation, allowing the tandem repeat sequences to serve as potential target for anticancer therapy [26]. In this study, a 9-AA peptides (APDTRPAPG) within the MUC1 tandem repeat sequence was employed as the target for aptamer selection, because it was identified as the most immunodominant peptide epitope within the protein core. MUC1 aptamers reported in previous studies was also selected using this epitope as target, presumably because the complete MUC1 protein core was difficult to obtain technically. The MUC1 aptamer MA3 developed in this study not only bound to the 9-AA MUC1 peptide epitope (Fig. 2A), but also recognized a 29-AA peptides (GSTAPPAGHGVTSAPDTRPAPGPGSTAPP) containing the entire MUC1 tandem repeat sequence (Fig. 3B). Moreover, MA3 exhibited potent binding to MUC1-positive cells A549 and MCF7, but relatively weak binding to MUC1-negative cells HepG2 and L02 (Fig. 4). The results suggest that MA3 may selectively recognize the MUC1 structure expressed on the surface of MUC1-expressing cancer cells.

In order for the aptamer to serve as targeting ligand, it needs to bind to MUC1 structure with certain specificity. In other words, the nonspecific binding to other proteins by the aptamer should be kept at minimum. Since albumin is the most abundant protein in blood, we evaluated the binding of albumin to MA3 and another MUC1 aptamer, S2.2, which had the highest targeting affinity among all the MUC1 aptamers reported in literature. While both MA3 and S2.2 bound to MUC1 with good affinity, the MA3's binding to albumin was significantly lower than that of S2.2 (Fig. 2). The data indicate that MA3 has a more selective binding to MUC1 with less nonspecific binding to albumin compared to S2.2, presumably because that the relatively larger size of MA3 (86-base for MA3 vs. 25-base for S2.2) potentially permits more complexity in its binding function, and that albumin was present in the buffer during the selection of MA3. Nevertheless, the *in vivo* MA3 binding specificity for MUC1 still needs to be evaluated with extensive future animal studies.

In this study, the uptake of doxorubicin by different cell lines was evaluated by confocal microscopy and flow cytometry. Free doxorubicin showed no preferential internalization in either MUC1-positive or MUC1-negative cells, presumably because the mechanism of uptake for free drug is similar for both cell lines (Fig. 7A and B). In contrast, Apt-Dox generated a doxorubicin fluorescence that was distinctively more potent in MUC1-positive cells as compared with that in MUC1-negative cells (Fig. 7C and D, 6E and F). This is a strong indication that Apt-Dox can discriminate efficiently between target and non-target cells.

The mechanisms of uptake of free Dox and Apt-Dox by cells appear distinct. Unlike free Dox, which exclusively stained the nuclei (Fig. 7A), Apt-Dox generated both nuclear and cytosolic staining (Fig. 7C). While free doxorubicin could non-selectively enter cells via passive diffusion, the polar components of the DNA aptamer presumably would prevent the doxorubicin intercalated in Apt-Dox from freely defusing into the lipid cell membrane. It was possible that Apt-Dox entered MUC1-positive cells via receptor-mediated endocytosis [27], which occurred after Apt-Dox bound to the MUC1 structure on A549 cell surface. Less entry of doxorubicin into HepG2 cells might come from lack of aptamer binding site on these MUC1-negative cells. As a result, a distinction in doxorubicin uptake between MUC1-positive and MUC1-negative cells was created. The cytotoxicity assay also supported the doxorubicin uptake data. For MUC1-positive cells, the cytotoxicity generated by Apt-Dox was similar to that generated by free doxorubicin; whereas for MUC1-negative cells, the cytotoxicity generated by Apt-Dox was reduced compared to that caused by free doxorubicin (Fig. 8, P<0.01). The data again suggested that the Apt-Dox could selectively deliver drug to MUC1-positive cancer cells. This presumably would decrease the adverse effects of doxorubicin against normal tissues that are largely MUC1-negative.

Many published studies on aptamer-guided targeted therapy employed nanoparticle (NP) as the carrier of anticancer agents [11], [28], [29]. However, it has also been reported that aptamer alone, without the carrier vehicle, could effectively deliver siRNA to tumor cells in vivo [30]. It would be interesting to know whether aptamer alone could also be employed for delivery of cytotoxic drugs (such as doxorubicin) to tumor cells. To achieve this goal, the drug needs to be associated with the aptamer, and the method for forming the aptamer-drug complex would probably influence the therapeutic efficacy. It is possible that covalent conjugation of doxorubicin to the tumor-targeting ligand may decrease the drug efficacy, presumably by altering the drug structure or by compromising the drug release from the targeting ligand [31]. Here in this study, the doxorubicin was not covalently conjugated to the aptamer, allowing the drug to be released more readily from the Apt-Dox complex. However, the in vivo stability of Apt-Dox complex is unknown at this stage, and extensive future research with in vivo animal studies is necessary to evaluate the stability of Apt-Dox in blood and tissues.

In summary, a novel MUC1 aptamer MA3 is developed in this study. The Apt-Dox complex could selectively deliver the cytotoxic agent doxorubicin to MUC1-positive cells, while reducing the drug uptake by MUC1-negative cells. Since MUC1 is over-expressed in most adenocarcinomas, the aptamer may have potential utility as a guiding ligand for targeted chemotherapy against these malignancies. Nevertheless, extensive future research with animal models is still needed to evaluate the *in vivo* binding specificity of the aptamer. In addition, it is also necessary to explore optimal ways to generate the aptamer-drug complex for most efficient *in vivo* delivery of cytotoxic anticancer agents to cancer cells.
